# Primary lesion of *Mucocutaneous Leishmaniasis* simulating external otitis

**DOI:** 10.5935/1808-8694.20120023

**Published:** 2015-11-20

**Authors:** Márcia dos Santos da Silva, Renato Telles de Sousa, Eucides Batista da Silva, Jorge Augusto de Oliveira Guerra, Nathália Matos Gomes, Renata Farias de Santana, Rebecca Souza Mubarac

**Affiliations:** a MD. 1^st^ year ENT Resident; b MSc in Otorhinolaryngology; Head of the Otorhinolaryngology Department - Getúlio Vargas University Hospital; c MSc in Infectology; Infectologist of the Amazon Tropical Medicine Foundation. Head of the Hospital Infection Control Committee - Getúlio Vargas University Hospital; d PhD in Infectology; Infectologist of the Amazon Tropical Medicine Foundation; e MD. 2^nd^ Year Dermatology Resident; f MD. 3^rd^ Year ENT Resident; g MD. 1^st^ Year General Practice Resident. Hospital Universitário Getúlio Vargas - Serviço de Otorrinolaringologia

**Keywords:** bacterial infections, leishmaniasis, cutaneous, otitis externa, staphylococcal skin infections

## INTRODUCTION

*Mucocutaneous Leishmaniasis* (MCL) has different clinical forms, depending on the leishmania species involved and its relation with the host^1^. In this paper, we report on the case of a patient, in whom the primary lesion was on the ear pinna, with bacterial infection associated.

## CASE PRESENTATION

**Figure 1:**
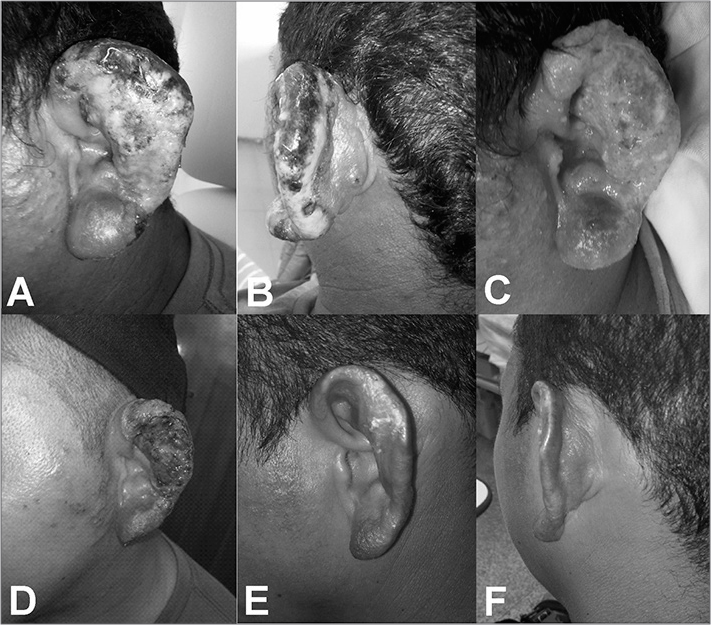
A: Suppurative lesion involving the ear pinna, with necrotic areas in the helix and, in a lesser degree in the anti-helix, infiltrative aspect in the lobe and papules in the pre-auricular region. Notice the lack of lesion in the external auditory canal inlet. B: Necrosis extending to the helix, infiltrative aspect in the adjacent skin and papule with central necrosis behind the ear, similar to the inoculation lesion. C: Granulomatous aspect seen in the bottom of the lesion after improvement in the suppurative process. Notice the persistence of papulae behind the year and the infiltrative aspect in the lobe and adjacent skin. D: Initial state of the ulcer healing process, about 20 days after treatment onset. E-F: Ear pinna reepithelialisation, 60 days after the end of treatment. Notice the improvement in the ear lobe and in the pre and retro auricular regions.

A 33-year-old male patient, coming from an endemic rural area, was referred to the ENT Department with a painful suppurative lesion in his left ear pinna, with necrotic areas in the helix and anti-helix, granulomatous aspect in the pre and retroauricular regions and an elevated papule with central necrosis, similar to an inoculation lesion (Figure 1A-B). The patient reported the signs and symptoms started about one month before, with intense pruritus, followed by papulae and ulceration in two weeks. Otoscopy was normal. Blood workup showed a leucocytosis of 14,498 cels/mm^3^ and ESR of 54 mm. Other laboratory parameters were normal. Mastoid and ear CT scans were also normal.

We surgically removed debris from the area, collected material for culture and biopsy, and started the patient on clindamycin. The the secretion culture isolated *Staphylococcus aureus*, and we decided to keep the antibiotic chose. After 14 days, the suppurative process regressed, making the granulomatous aspect clearer in the bottom of the ulcer, infiltrating the adjacent skin (Figure 1C).

Histopathology reported an intense inflammatory infiltrate, made up of lymphocytes, plasmocytes, eosinophilic granulocytes and cellular debris on the dermis.the findings were suggestive of MCL; However, no parasite was seen. We then roughed up the borders of the lesion for a direct exam, and then we found the leishmania in the amastigote form.

The patient was then started on Antimonate (15mg/kg/day) for 30 days. After 20 days of treatment, the granulomatous process regressed and the ear pinna started to reepithelialise (Figure 1D). About 60 days after the end of treatment, the ear pinna was completely reepithelialised (Figure 1E-F).

## DISCUSSION

The classic MCL is a well-outlined ulcer, with elevated borders and granulomatous bottom, which sprouts out at the place of inoculation^2^. The secondary infection may happen in 54.2% of the patients, and the most commonly found germ is the *Staphylococcus aureus*^3^, the same found in our case, which explains the good initial response after starting the clindamycin.

Clinical suspicion was based on associating lesion appearance with epidemiological data^4^. The characteristic aspect of the leishmaniasis ulcer was only seen after controlling the suppurative process, when the granulomatous bottom became more evident, and the diagnosis of MCL was reinforced by the patient's epidemiological past.

Finding the *Leishmania* is the gold standard to diagnose MCL. Histopathology can find amastigotes in only 25% of the skin lesions. In the other cases, findings may suggest the diagnosis, but not define it^5^. Direct microscopy has a sensitivity ranging between 50 and 70%^4^. In the case hereby presented, histopathology suggested MCL, and the parasites were found by direct exam, thus defining the diagnosis.

The drug-of-choice to treat all types of leishmaniasis are pentavalent antimonials. In Brazil, the Department of Health recommends the dose of 15 mg/Kg/day, for 20 days for the skin lesions^5^, which is what we used in the patient hereby reported, and we extended it for 30 more day because of the severity of the lesion. When there is no response to treatment, the drugs of choice are the pentamidines and amphotericin B. Alternative treatments, such as azithromycin, paramomycin, miltefosine, pentoxifylline, allopurinol, fluconazole e itraconazole are still not proven in large scale^5^^,^^6^.

## FINAL REMARKS

MCL lesions have an intense polymorphism. The patient's epidemiological past and a high degree of suspicion are fundamental for proper diagnosis.
